# Hygiene and Sunday

**Published:** 1890-02

**Authors:** 


### Hygiene and Sunday.

Among the questions treated of at the recent congresses at Paris, that of the observance of the Sabbath as a day of rest was not the least interesting. The congress on this subject was presided over by M. Leon Say, who remarked that this rest, which several religions rendered obligatory, is a law of nature, and consequently a law of hygiene, the excellence of which has long been demonstrated, although it is not to be found in all national codes. The resting on the seventh day is of Biblical origin, and the custom of counting the days by seven was formerly the rule among the most divease races—in India, as among the Celts, in China as well as in Arabia. Now that hygiene has become a positive science, it confirms the moral and material necessity for a temporary rest on the seventh day. The idea was adopted in principle by all the members of the congress, which received the patronage of two political celebrities—Mr. Harrison, President of the United States of America, and Mr. Gladstone. In a letter which was read publicly, President Harrison declared that he considered that all workers, whether with the hands or with the head, were in need of rest, which alone can guarantee the general observance of the Sabbath. Mr. Gladstone declared that he attributed his robust health and his longevity to his invariable observance of the Sabbath rest. Several reports were presented to the congress, and physicians, professors, philosophers, and hygienists are in accord on this point. All, without exception, support for workers of all classess and of all ages a weekly day of rest, which should even be made obligatory. It may here be noted that in 1881 this subject was open to competition by the Swiss Government for a prize, which was awarded to Dr. Niemeyer, of Leipsic. The subject was brilliantly treated by Dr. Niemeyer, who observed that the Dominical rest is the first commandment of hygiene, which should be followed to obtain a peaceful and continued amelioration of society, and in this respect it is as much a rational institution as a religious one. The following is the summary of the conclusions voted by the great majority of the members of the congress: "Rest on Sunday is possible in varying degrees in all industries. Sunday is the day which best suits the employer and employed, both as regards the individual himself and his family, and it is well that the day of rest should be, as much as possible, the same for all. When the Sunday rest is impracticable for certain reasons, it should be replaced by some other day, so that the workman may have fifty-two days' rest in the year as equally divided as possible. This rest permits man to produce considerably more and better work, inasmuch as it contributes to maintain his zeal and to restore his physical forces."—*Lancet*, Nov. 23, 1889.

